# Building a breast cancer detection and treatment platform in the Democratic Republic of the Congo by integrating training, service and infrastructure development

**DOI:** 10.3332/ecancer.2021.1233

**Published:** 2021-05-13

**Authors:** Kabongo Mukuta Mathieu, Tankoy Gombo YouYou, Michael L Hicks, Alex Mutombo, Mukanya Mpalata Anaclet, Mulumba Kapuku Sylvain, Leeya Pinder, Maya M Hicks, Louis Kanda, Mirielle Kanda, Groesbeck P Parham, Ronda Henry-Tillman

**Affiliations:** 1 Biamba Marie Mutombo Hospital, No. 9777, Boulevard Lumumba, Commune de Masina, Kinshasa, Democratic Republic of the Congo; 2 Department of Obstetrics and Gynecology, University of North Carolina at Chapel Hill, 101 Manning Dr, Chapel Hill, NC 27514, USA; 3 Department of Obstetrics and Gynecology, University Teaching Hospital – Women and Newborn Hospital, 10101 Nationalist Way, Lusaka, Zambia; 4 St Mary Mercy Cancer Center, 36475 Five Mile Rd, Livonia, MI 48154, USA; 5 St Joseph Mercy Oakland Cancer Center, 44405 Woodward Ave, Suite 202, Pontiac, MI 48341, USA; 6 McLaren Macomb Medical Center, 1000 Harrington Blvd, Mount Clemens, MI 48043, USA; 7 Department of Oncology, University of Washington, 1959 NE Pacific St, Seattle, WA 98195, USA; 8 Howard University College of Medicine, 520 W St NW, Washington, DC 20059, USA; 9 Dikembe Mutombo Foundation, 400 Interstate N Pkwy, Suite 1040, Atlanta, GA 30339, USA; 10 Winthrop P Rockefeller Cancer Institute, University of Arkansas for Medical Sciences, 4301 West Markham St, Slot #725, Little Rock, AR 72205, USA; a https://orcid.org/0000-0002-1819-155X; b https://orcid.org/0000-0002-8929-7810; c https://orcid.org/0000-0002-1993-3367; d https://orcid.org/0000-0002-1782-9523

**Keywords:** breast cancer, capacity building, cancer control in Africa, woman’s breast cancer center in Africa, Democratic Republic of Congo, surgical training in LMIC

## Abstract

**Background:**

Breast cancer is a leading cause of cancer-related morbidity and mortality in sub-Saharan Africa, a global region where opportunities for breast care of any type are extremely limited due to insufficient infrastructure, a paucity of clinical services and vast shortages of trained human resources.

**Methods:**

A team of Zambian and US gynaecologic and breast oncology experts and nurse-specialists made multiple visits (each lasting 5 working days) to the Democratic Republic of the Congo (DRC), over a 2-year period. During each of five week-long site visits, hands-on training of local Congolese health providers was conducted during which time they were taught clinical breast exam (CBE), breast and axillary ultrasound, ultrasound-guided core needle biopsy/fine needle aspiration (FNA) and breast surgery. Simultaneous with the training exercises, a new breast care clinic was established and operationalised, and existing surgical theatres were upgraded. All activities were implemented in a private sector health care facility – Biamba Marie Mutombo Hospital – in the capital city of Kinshasa.

**Results:**

From April 2017 to August 2020, a total of 5,211 women were identified as having breast abnormalities on CBE. Ages ranged from 26 to 86 years; median age: 42.0 (±14.1) years. Ultrasound abnormalities were noted in 1,420 (27%) clients, of which 516 (36%) met the criteria (indeterminate cystic lesion, solid or suspicious masses) for ultrasound-guided core needle biopsy or FNA. Pathology reports were available for 368 (71%) of the 516 clients who underwent biopsy, of which 164 were malignant and 204 benign. The majority (88%) of the cancers were advanced (TNM stages 3 and 4). Surgical procedures consisted of 183 lumpectomies, 58 modified radical mastectomies and 45 axillary lymph node dissections. Clinical competency for diagnostic and surgical procedures was reached early in the course of the training programme.

**Conclusion:**

By integrating onsite training with simultaneous investments in clinical service and infrastructure development, the barriers to breast cancer diagnosis and treatment were disrupted and a modern breast care service platform was established in a private sector health care facility in the DRC.

## Background

According to the most recent global cancer statistics provided by the International Agency for Research on Cancer (Globocan 2020), breast cancer is the most commonly diagnosed cancer in the world, in particular due to high prevalence in low- and middle-income settings [1]. It is predicted that over the next two decades, 70% of all new breast cancers will occur in resource-constrained environments [2]. The paucity of diagnostic and therapeutic infrastructure and oncology-specific human resources in Africa has been thoroughly documented [3, 4–6]. Of particular note are the severe shortages of pathologists [7, 8], medical oncologists [9, 10], surgeons [11] and radiation oncologists [12]. All of the aforementioned deficits result in limited access to diagnosis and treatment, and decreased survival. For instance, it has been estimated that of the 15 million cancer cases that occurred by the end of 2015, approximately 80% required surgical oncologic care, and 75% of these (mostly in LMICs) did not receive safe, affordable and timely surgical services [11]. Most of the chemotherapeutic agents on the World Health Organization’s Essential Medicines List, used in the treatment of breast cancer, remain largely unavailable or unaffordable for patients in many sub-Saharan African (SSA) countries [13], thereby restricting opportunities for the use of recommended neoadjuvant and adjuvant systemic therapy. A recent systematic review found that 29 countries in Africa lack any radiotherapy capacity [12], limiting access of women in these countries to adjuvant radiation therapy, a standard form of treatment for locally advanced breast cancer [14]. In the first large-scale study of women (*n* = 2,156) with breast cancer from six African countries, 3-year overall survival was only 50% (95% CI: 48–53) [15]. A contributing factor was prolonged delay in acquiring a diagnosis after presentation to a health care facility [16], a direct manifestation of the lack of cancer care infrastructure. In summary, while screening mammography combined with timely, high-quality diagnostics and management (surgery, hormonal therapy, biologics, chemo-radiation) has significantly improved breast cancer outcomes in high income countries [17], such services are scarce in low- and middle-income countries (LMICs).

This tragic, deplorable and inhumane circumstance finds its most concrete expression in fragile, conflict and violence affected societies, like the Democratic Republic of the Congo (DRC). The DRC is the world’s largest fragile, conflict and violence affected nation, and is home to a population of 89 million people, of which the vast majority (~70%) live on less than $1.90 a day [18]. In a recent (2020) country profile, cancers of the breast (incidence 12.6%/mortality 8.9%) and cervix (incidence 11.8%/mortality 12.7%) were reported as the most commonly diagnosed cancers and cancer-related causes of death among the 48,890 cancers and 36,691 cancer-related deaths recorded in 2018 [19]. Although national strategic guidelines have recently been developed [20], public sector programmes for cancer prevention, screening and management are absent. Neither is there a professional oncology workforce [21, 22]. It is against this background and within this context that we embarked on an effort to disrupt the status quo and establish women’s cancer care services within the country.

## Methods

Our major objective was to increase access to breast care services for women, as part of a broader effort to build a women’s cancer centre in the DRC. Our approach consisted of integrating training with service delivery and infrastructure development. As a first step, we convened a meeting with officials from the Ministry of Health of the DRC, at which time we presented our intentions, goals and approach. After receiving Ministry of Health (MOH) clearance, we proceeded with the educational, service delivery and infrastructure development activities, as planned.

### Situational assessment

As part of the implementation strategy, a multi-sectoral consensus conference was held to assess and document the status of clinical breast examination (CBE), breast ultrasound, ultrasound-guided core needle biopsy/fine needle aspiration (FNA), surgery, chemotherapy, radiation and pathology services within the country, as well as the presence of any designated cancer care facilities and programmes. The results of the assessment highlighted the significant workforce, infrastructure and funding gaps in the public sector, across all areas of cancer control. Informed by this evidence, our team of US and Zambian oncologists and nurse-specialists worked collaboratively with a team of local Congolese physicians and nurses to design a training curriculum and determine the clinical infrastructure required to address the major aforementioned deficiencies. The designated site for implementation of the breast care service platform was the Biamba Marie Mutombo Hospital (BMMH), a private sector 150-bed general hospital with a public health care focus, located in the capital city of Kinshasa. Opened in 2007, patients are charged on a sliding scale and no one is turned away due to an inability to pay. Partnerships with other non-profit organisations and the generosity of private contributors help ensure the sustainability of the facility. The decision to implement training activities and clinical/surgical services within a private sector facility was based on recommendations put forth by the consensus conference. It was felt this approach could facilitate programme initiation and sustainability by circumventing many of the logistical problems present in the public sector, such as lack of personal security, bureaucratic inefficiencies, unreliable procurement systems and the potential for politicisation and militarisation of health services.

### Training

A team of Zambian and US gynaecologic and breast oncology experts and nurse-specialists made multiple visits (each of 5 working days duration) to the DRC, over a 2-year period. The training activities occurred during each day of the 5-day visits, consisting of a combination of didactics and hands-on instruction, led by a US breast surgical oncologist (master trainer). Women attending the newly opened cervical cancer ‘screen and treat’ clinic at BMMH were asked about breast symptoms, and if present their willingness to have a CBE. Those answering in the affirmative underwent a CBE by the cervical cancer screening nurses, all of whom who had been crossed trained to perform CBE. If discovered to have abnormalities (masses, thickening, new onset asymmetry, pain, nipple discharge, etc.), they were immediately referred to the newly established breast clinic where they underwent evaluation by trainees. In accordance with the MOH-approved competency-based training curriculum, BMMH staff physicians, nurses and physician assistants were trained in the following areas: (1) breast self-awareness; (2) CBE; (3) smart phone and traditional breast ultrasound; (4) axillary node ultrasound for the evaluation of suspicious axillary nodes; (5) ultrasound-guided FNA and ultrasound-guided core needle biopsy of breast masses; and the surgeon only in (6) surgical treatment of breast cancer (breast lumpectomy, modified radical mastectomy, axillary node dissection), (7) breast oncoplastic surgery and (8) palliative breast surgery.

The curricula for breast imaging were designed for both smart and standard breast ultrasonography, adopted from courses taught by the American College of Surgeons [23]. Trainees were instructed utilising didactics, simulated and live models. Didactics consisted of ultrasound physics, basic principles of ultrasound, utilisation of standard ultrasound and smart device components with the associated linear transducers. Instructions on scanning techniques were performed using both phantom and live breast models, including recognition of the characteristics associated with ductal anatomy, normal breast parenchyma, benign versus malignant focal breast masses, as well as the axillary and axillary nodal anatomy. FNA for cystic lesions and suspicious axillary adenopathy, and percutaneous core needle biopsy for solid breast masses utilising the spring-loaded core needle biopsy device were demonstrated on phantom models. Understanding of local anaesthetics was reviewed. Trainees were instructed on insertion of core biopsy needles and obtaining five core samples. Following completion of the training curricula, trainees were tested and checked off on the ability to demonstrate their understanding of the physics and principles of breast ultrasound, breast ultrasound scanning techniques, recognition of normal and abnormal findings as well as demonstration of the ability to recognise and aspirate a fluid collection (cyst or seroma) and perform a percutaneous biopsy utilising the spring-loaded device. Other aspects of training consisted of a detailed review of pathology and its implications relative to patient management, as well as concordances and discordances of ultrasound findings and pathology results.

Specific to the surgical procedures, preoperative video teaching sessions took place prior to each procedure. The various types of procedures were reduced to their most critical and difficult constituent parts, then taught through active bedside mentoring in the surgical theatre (mastectomies, axillary node dissections). Each teaching case consisted of the trainee performing the procedure under the guidance of the master trainer, with relevant questioning and explanation of the rationale behind each step performed during the procedure. Throughout each 5-day training period, the trainee was asked to perform, on his own, a mental narrative of the procedures several times a day, reviewing the rationale behind each step and mentally visualising potential intra-operative complications and corrective responses. Post-surgical debriefing of the operation was conducted consistently. Onsite clinical training was supplemented between visits with virtual clinical mentorship. Lumpectomies performed for cancer treatment were carried out on small lesions (<3 cm) with benign ultrasound findings of the axilla, and in some cases for larger malignant lesions in patients completely opposed to mastectomy. Patients who underwent lumpectomy for cancer were counselled on the higher risk of local recurrences due to the lack of radiation therapy facilities in the DRC [24–26]. Modified radical mastectomy was performed on all patients with malignant breast lesions greater than 5 cm, or multicentric tumour locations, and in whom there were obvious nodal metastasis or clinically suspicious axillary nodes on exam and/or axillary node ultrasound. Axillary node dissections were carried out in most cases for reduction of tumour burden, since the majority of patients had advanced disease and radiation therapy was unavailable [27]. Post-operative rounds were conducted twice a day, prior to the beginning of surgery and afterwards, under the leadership of the surgical trainees and supervision of the master trainer.

Written consent in the commonly used languages of English, French, Lingala or Swahili was obtained prior to clinical or surgical procedures, including biopsies. In case of illiteracy, oral consent was obtained. Every consent required a witness. The proficiency of the trainees was determined through direct observation of their skills in performing the various procedures by the US breast surgical oncologist. Clinical competency was defined as a trainee being able to perform any of the stated procedures correctly and independently, as judged by the master trainer.

### Clinical services and infrastructure development

Targeted investments in equipment and supplies plus modifications of existing physical structures within the hospital were used to create the necessary clinic space and upgrade the surgical theatres in which training activities took place, and clinical services were simultaneously provided.

## Results

**Figure 1.**
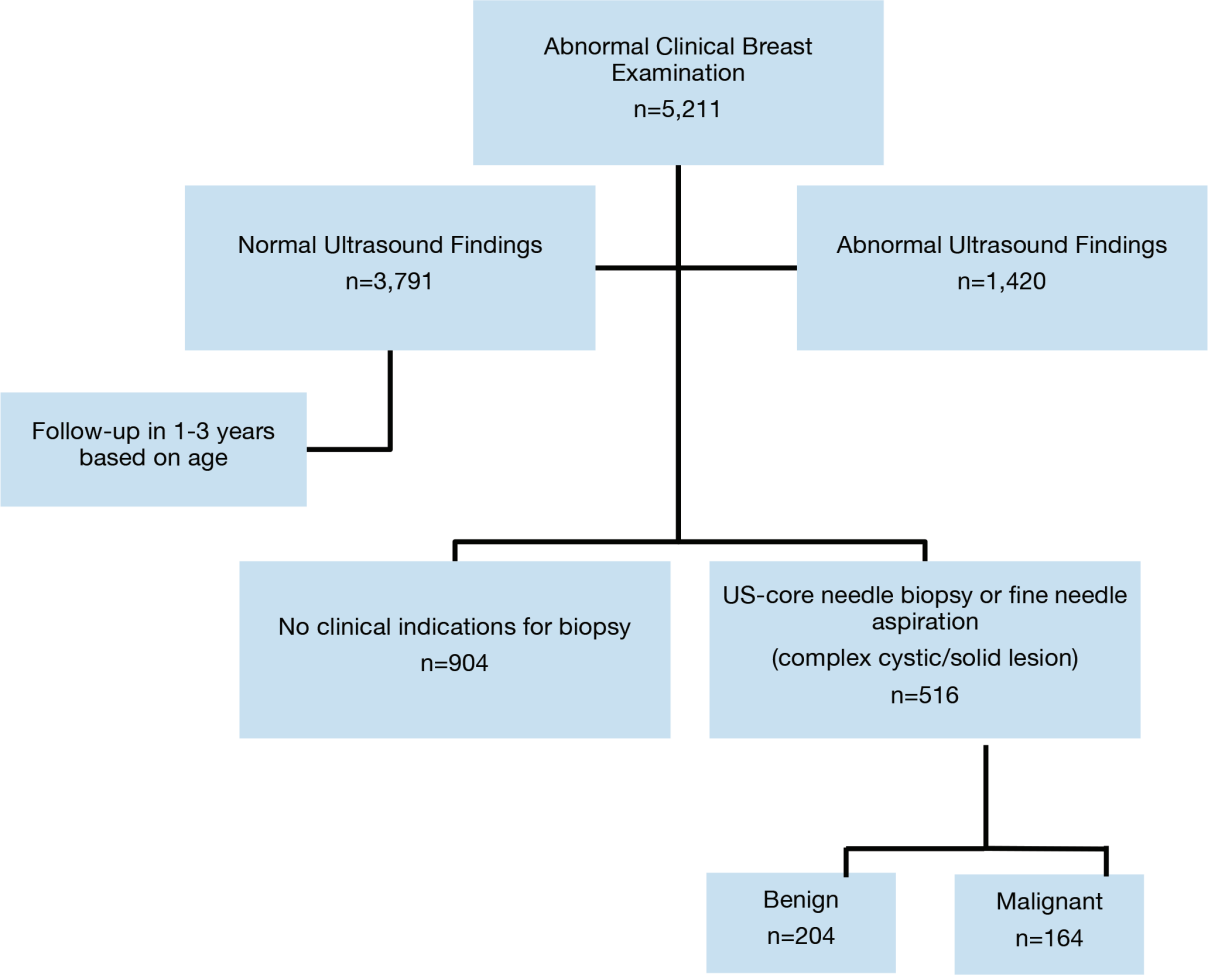
Clinical algorithm.

**Figure 2.**
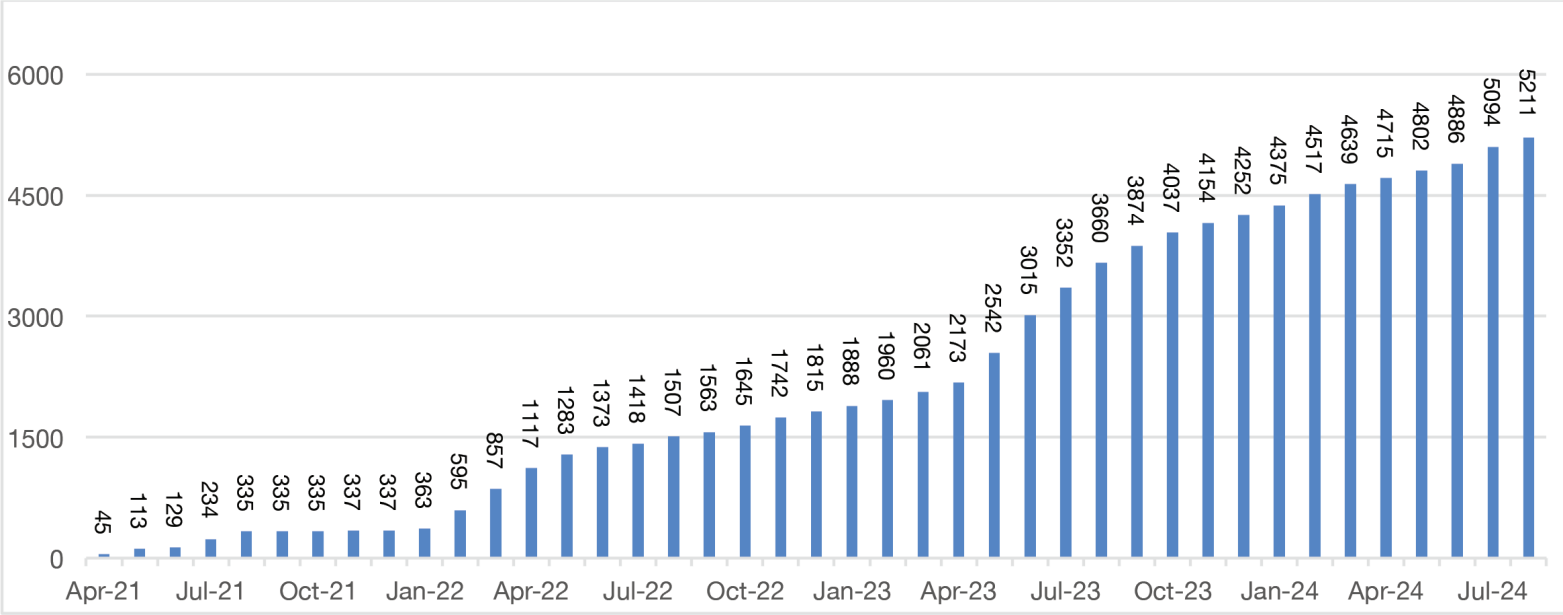
Cumulative number of women screened for symptomatic breast disease (n = 5,211).

**Figure 3.**
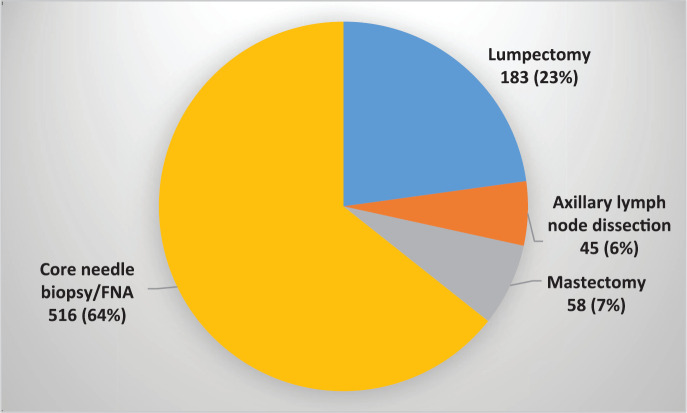
Diagnostic and surgical procedures (n = 802).

**Figure 4.**
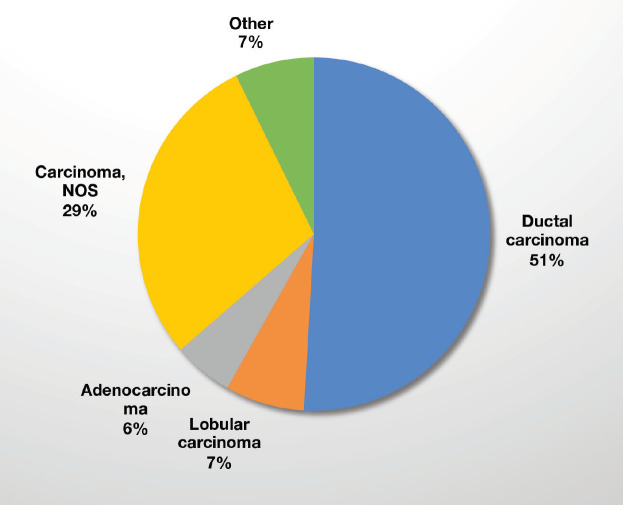
Malignant tumour types (n = 164).

From April 2017 to August 2020, a total of 5,211 patients were identified as having a breast abnormality on CBE (Figure 2). Ages ranged from 6 to 86 years; median age: 42.0 (±14.1) years. Ultrasound abnormalities were noted in 1,420 (27%) patients, of which 516 (36%) met the criteria (indeterminate cystic lesion, solid or suspicious masses) for ultrasound-guided core needle biopsy or FNA (Figure 1). Pathology reports were available on 368 (71%) of those who underwent biopsy, of which 164 were malignant and 204 benign (Figure 1). The majority (~90%) of the cancers were advanced (TNM stages 3 and 4) (Table 1). The remainder were stage 2. Approximately half (51%) of the malignancies were ductal carcinomas. The distribution of histology types is shown in Figure 4. The benign lesions were mostly fibrocystic masses and fibroadenomas. Surgical procedures consisted of 183 lumpectomies, 58 modified radical mastectomies and 45 axillary lymph node dissections (Figure 3). All cancer cases were referred to the newly developed chemotherapy unit at BMMH for evaluation.

Two patients had oncoplastic surgery and five underwent purely palliative resections for quality of life reasons, i.e., large lesions associated with significant pain, incapacitating their ability to ambulate and maintain hygiene.

There were no reported complications with any of the diagnostic procedures. There were seven cases of wound dehiscences – seromas with small surgical site skin separation (grade 1 surgical complications) – in our surgical population [28].

Figure 3 shows the numbers and types of diagnostic and surgical procedures performed during the course of the training.

## Discussion

**Figure 5.**
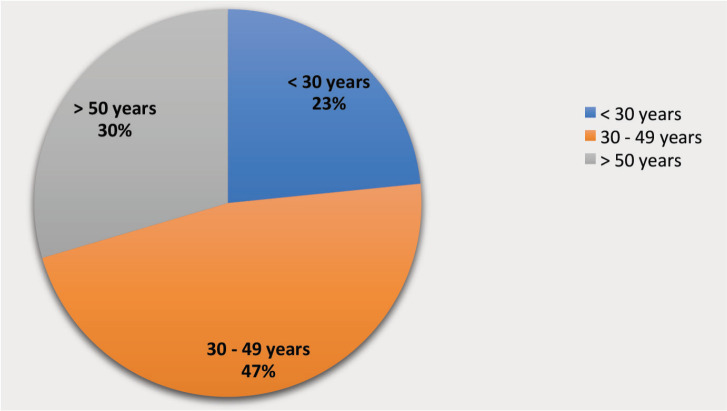
Age distribution of women screened for symptomatic breast disease (n = 5,211).

Analysis of our results reveals that the majority of women evaluated with CBE were between the ages 30 and 49 years with a median age of 42 years (Figure 5). This is generally consistent with other published reports of CBE in SSA, a region where 25% of patients diagnosed with breast cancer are between 30 and 45 years of age, compared to 10% in high income countries [27, 29–32].

Of the 5,211 patients with abnormalities on CBE, 516 (10%) had findings that met the criteria warranting a biopsy. This number is in line with other reports from SSA that report positive findings on 10%–13% on breast ultrasound that require biopsy [29–31]. Although the numbers of patients in our cohort with CBE abnormalities were larger than other published reports from the African region, when criteria for biopsy were restricted to indeterminate cystic lesions, solid or suspicious masses, only 10% of those who underwent ultrasound met the criteria for biopsy.

In an earlier breast cancer study in the DRC, participating women were initially investigated with CBE. Those in whom a palpable mass was detected were referred to the hospital where they received a mammography and ultrasound and – in case of suspicious findings – a core needle biopsy. Compared to our cohort, theirs was smaller (*n* = 4,315 versus *n* = 5,211), had fewer patients with indications for biopsy 133/497 (27%) versus 516/1,420 (36%) and a lower yield of malignancies among those who were initially evaluated with CBE (100/4,315) 2.3% versus (164/5,211) 3.1%. Unlike our approach, they did not address skills transfer, infrastructure development or treatment [32]. Other studies that have used CBE followed by ultrasound-guided core needle biopsies had much smaller numbers of patients than ours, with malignancy yields ranging from 5%–64% in patients meeting ultrasound criteria for biopsy; our rate was 45% [29–31]. Despite their differences, these studies validated the efficacy, efficiency and portability of ultrasound as a tool convenient for identifying breast lesions that require biopsy in a LMIC setting. Based on our review of the literature, our series of ultrasound-guided core needle biopsies (*n* = 516) on women initially evaluated with CBE is the largest reported from the continent of Africa [29–32].

Clinical competency for diagnostic and surgical procedures was reached early in the course of the training programme. Competency in identifying breast masses via ultrasound and performing ultrasound-guided core needle biopsies and FNA occurred after 46 cases. Surgical competency for performing lumpectomies was reached after five cases; modified radical mastectomies and axillary node dissection after ten cases. The latter occurred after the first site visit and permitted the trainee to begin performing these procedures independently, between site visits.

The method of surgical skills transfer we used for workforce capacity building in breast care has previously been shown to be effective in surgical oncology capacity building cervical cancer in other African settings [33]. The skills transfer approach we used facilitated the performance of 183 lumpectomies (178 cases above competency), 58 modified radical mastectomies and 45 axillary node dissections (48 and 35 cases above competency, respectively). The cases performed above competency without serious complications by the newly trained surgeon may be due to the strength and efficacy of the training methodology but may equally be a reflection of the learning ability of the trainee and/or his prior surgical experience. To the author’sknowledge, this is the largest report of surgical oncology capacity building in the DRC for breast cancer. With the global rise in cancer cases and the estimation that 80% will require surgery at some point during the course of the disease [11], surgical capacity building, especially in LMICs is of utmost importance.

In our series, only 10 (12%) patients had early-stage disease (Table 1). All had small lesions (2 cm or less) that were evaluated with ultrasound-guided core needle biopsy, and histologically confirmed. They also underwent axillary node assessment with ultrasound to rule out the presence of features suggestive of lymph node metastasis, using criteria of cortex thickness of 3 mm or greater and fatty hilum reduction [34]. These cases were identified later in the course of the training programme, possibly reflecting improvement in the ability of the trainees to identify smaller lesions on ultrasound. They could also represent women beginning to present to the healthcare facility with early symptoms.

Consistent with the current literature on breast cancer in SSA, the majority of our patients had advanced stage disease (Table 1) and the most predominant malignant cell type was invasive ductal carcinoma [17, 29–32] (Figure 4). All patients with histologically confirmed breast cancer were referred to the newly established chemotherapy unity at BMMH for evaluation and treatment. Additionally, they were all placed on Tamoxifen therapy empirically, at the time of diagnosis. Several studies have highlighted more aggressive triple negative breast cancers in SSA [35, 36]. However, a recent systematic review and meta‐analysis demonstrated a predominance of better prognosis oestrogen‐receptor‐positive tumours in SSA [37]. Recent findings from the African Breast Cancer-Disparities in Outcomes study of SSA women breast cancer confirm that triple negative tumours make up less than one‐fifth of cancers [38]. We are aware of the risk profile of hormonal therapy [39]. However, we had very limited capacity to direct hormonal therapy based on tumour receptor status secondary to not having a hospital-based pathology service, and the cost prohibitive nature of immunohistochemical stains at outside labs. Thus, our management options were based on the accumulated evidence of receptor status of women with breast cancer in SSA [37, 38], the contextual circumstances and our professional opinion of how we could benefit the most patients when faced with these limitations. The need for in-house pathology services accelerated the present collaboration between BMMH and the American Society of Clinical Pathology which is presently building pathology infrastructure at BMMH, inclusive of immunohistochemistry.

On the fourth visit, the master trainer instructed the trainee how to perform oncoplastic resections utilising the Batwing technique [40]. This is in alignment with our surgical skills transfer approach which includes the ability of surgical trainees to perform palliative surgeries to improve the quality of life for patients with very large tumours that prevented ambulation and proper hygiene secondary to their exophytic, fungating nature. The five palliative cases (toilet mastectomies) performed by trainees required removal of significant amounts of chest muscle and skin, resulting in the need to perform skin flap advancements for closure. Palliative surgery in regions with high rates of advanced breast cancer is an essential surgical modality for compassionate resolution of suffering and restoration of human dignity.

## Conclusion

We report the successes, challenges and preliminary outcomes of developing breast detection and treatment services in the first women’s cancer centre in the DRC. Implementing a breast care service platform at a private sector healthcare facility (BMMH) has provided access to care for symptomatic women. It has also allowed local Congolese health care providers the opportunity to accumulate experience in the diagnosis and treatment of breast cancer in one facility, thereby increasing their expertise. Local capacity now exists for the detection, diagnosis and surgical management (breast conservation, radical resection and palliation) of breast cancer in Kinshasa, DRC. This established cancer care service platform can now be leveraged to build workforce capacity in the public sector through future public–private partnership training programmes.

Our effort represents a clear demonstration of how to improvise in a resource-constrained setting in order to initiate cancer care services. Enhancement, expansion and perpetuation of this approach throughout the DRC and other SSA countries have the potential for improving breast cancer care throughout the African continent [41, 42] When combined with widespread health promotion, breast cancer awareness and CBE, it may lead to downstaging of disease and possibly a reduction in mortality [43]. Involving the private sector allows for swiftness, provides a one stop shop and less bureaucracies.

Future plans are to fully integrate pathology services (including immunochemistry) into the clinical care platform, recruit additional general surgeons and other healthcare personnel for training from the DRC and other Francophone African countries, expand the chemotherapy unit and establish more rigorous quality assurance systems. Establishment of a tissue bank and involvement in relevant research is also of critical importance to improving outcomes. These efforts will be carried out within the larger vision of developing BMMH into a regional centre of excellence for women’s cancer control.

## Conflicts of interest

None of the authors declare any conflicts of interest.

## Funding statement

Funding for the initiative was provided by a generous grant from the Howard G. Buffett Foundation whose mission is to catalyse transformational change to improve the standard of living and quality of life, particularly for the world’s most impoverished and marginalised populations.

## Figures and Tables

**Table 1.** Clinical stage distribution of invasive breast cancers at diagnosis (n = 85).

Clinical stage	n (%)
Stage 0	0
Stage I	0
Stage II	10 (12%)
Stage III	30 (35%)
Stage IV	45 (53%)
